# Sequencing bilateral and unilateral task-oriented training versus task oriented training alone to improve arm function in individuals with chronic stroke

**DOI:** 10.1186/s12883-014-0236-6

**Published:** 2014-12-14

**Authors:** Sandy McCombe Waller, Jill Whitall, Toye Jenkins, Laurence S Magder, Daniel F Hanley, Andrew Goldberg, Andreas R Luft

**Affiliations:** School of Medicine, Department of Physical Therapy and Rehabilitation Science, University of Maryland, 100 Penn Street, Baltimore, MD 21201 USA; Department of Epidemiology and Public Health, University of Maryland, 660 W. Redwood Street, Baltimore, MD 21201 USA; Acute Care Neurology, Director, Division of Brain Injury Outcomes, The Johns Hopkins Medical Institutions, 1550 Orleans Street, Baltimore, MD 21231 USA; Geriatric Research, Education and Clinical Center (GRECC), Baltimore VA Medical Center, University of Maryland School of Medicine, 10 North Greene Street, BT/18/GR, Baltimore, MD 21201 USA; Department of Neurology, University and University Hospital Zurich, Frauenklinikstrasse 26, Zürich, 8091 Switzerland

**Keywords:** Stroke, Bilateral arm training, Upper extremity, fMRI, Sequential training

## Abstract

**Background:**

Recovering useful hand function after stroke is a major scientific challenge for patients with limited motor recovery. We hypothesized that sequential training beginning with proximal bilateral followed by unilateral task oriented training is superior to time-matched unilateral training alone. Proximal bilateral training could optimally prepare the motor system to respond to the more challenging task-oriented training.

**Methods:**

Participants: Twenty-six participants with moderate severity hemiparesis Intervention: Participants received either 6-weeks of bilateral proximal training followed sequentially by 6-weeks unilateral task-oriented training (COMBO) or 12-weeks of unilateral task-oriented training alone (SAEBO). A subset of 8 COMB0 and 9 SAEBO participants underwent three functional magnetic resonance imaging (fMRI) scans of hand and elbow movement every 6 weeks. Main Outcome Measures: Fugl-Meyer Upper extremity scale, Modified Wolf Motor Function Test, University of Maryland Arm Questionnaire for Stroke, Motor cortex activation (fMRI).

**Results:**

The COMBO group demonstrated significantly greater gains between baseline and 12-weeks over all outcome measures (p = .018 based on a MANOVA test) and specifically in the Modified Wolf Motor Function test (time). Both groups demonstrated within-group gains on the Fugl-Meyer Upper Extremity test (impairment) and University of Maryland Arm Questionnaire for Stroke (functional use). fMRI subset analyses showed motor cortex (primary and premotor) activation during hand movement was significantly increased by sequential combination training but not by task-oriented training alone.

**Conclusions:**

Sequentially combining a proximal bilateral before a unilateral task-oriented training may be an effective way to facilitate gains in arm and hand function in those with moderate to severe paresis post-stroke compared to unilateral task oriented training alone.

## Background

The recovery of useful hand function after stroke is a major clinical challenge for individuals with moderate to severe paresis. Progress in improving hand function has been demonstrated in participants with substantial residual function using task-oriented constraint-induced therapy (CIT), however such results do not occur in participants with more severe limb impairment (i.e. upper extremity Fugl-Meyer scores below 25) [1-6]. Studies targeting more severe populations show promising results but are limited to proximal training approaches and do not result in improvements in paretic (P) hand function [7-11]. Innovative training strategies targeting active rehabilitation of hand and arm function for those with moderate to severe hemiparesis are needed. A single training approach may not optimally address this need. We proposed that sequencing proximal bilateral and unilateral task-oriented arm training strategies would provide an improvement over a single approach and reach a wider severity range of participants.

It is well established that active use of the limb in a goal directed manner is essential for functional gains. However, in more impaired participants, two issues limit the active involvement of the arm and hand in training: 1) reduced proximal arm function to transport the hand to a target, and 2) inability to actively open and close the hand once at the target. This is not to say recovery progresses from proximal to distal but that both actions are necessary for functional arm use. To address these specific challenges we developed a two-phase sequential rehabilitation approach. In Phase 1, participants received progressive bilateral arm training with rhythmic auditory cueing (BATRAC) for 6-weeks with a focus on improving proximal motor function and to potentially “prime” the central nervous system before transitioning in Phase 2. In Phase 2 participants received unilateral task-oriented training using the Saeboflex dynamic hand orthosis to aid in active participation of the hand. We compared this training approach to two 6-week sessions of time-matched unilateral task-oriented Saeboflex training with no proximal movement priming. Based on our previous work involving BATRAC training, we observed bilateral brain activation following bilateral arm training as well as arm transport gains. Studies of those who are well recovered from stroke show that initial bilateral activations seen acutely resolve to contralateral brain activation as functional recovery occurs. In individuals with chronic stroke of moderate to severe impairment, nonparetic arm use dominates for functional task performance; activities that would not stress activation of the lesioned hemisphere. In our conceptual framework we sought to provide a training that could potentially activate both lesioned and non lesioned hemispheres initially and engage the paretic arm in basic transport motions and then progress to a unilateral training approach that built upon basic arm transport to include grasp and release components. The transition from bilateral to unilateral training was also to emphasize the lesion hemisphere activation. Therefore, we were investigating if a sequenced two-step training approach yielded better gains than one that involved transport and manipulative training from the start and only trained the paretic arm.

We investigated the brain activation patterns of participants through functional magnetic resonance imaging (fMRI) for both elbow and hand movements in a subset of participants who were eligible for the testing. fMRI is a surrogate marker for training-induced behavioral changes and may provide indications of the neural mechanism of a specific therapy [12-14]. Several studies have indicated lasting changes in motor control networks that are reflected in altered brain activation patterns during movement of the P limb. These changes have been observed in cortical (sensorimotor cortices, premotor cortex, cingulate cortex, parietal and frontal regions) and subcortical areas (cerebellum, brainstem) [15]. We hypothesized that the sequential combination training would be superior to the single focus training in producing behavioral gains in upper extremity (UE) functional outcomes and that imaging results in our subset of participants would show different neural mechanisms associated with the two training approaches with cortical and subcortical activation increases (in sensorimotor and premotor cortex and cerebellum) in the sequential training group alone.

## Methods

### Subjects and testing

Thirty subjects with chronic hemiparesis from unilateral stroke were enrolled in this study with 26 completing. After determination of eligibility, subjects were randomly assigned to training group using a block allocation system that was concealed to investigators. Four withdrew due to personal health issues. All subjects had moderate to severe impairment based on initial score on the Fugl–Meyer Upper Extremity Test (FM) (ranging from 9–35). Inclusion criteria included chronic unilateral ischemic or hemorrhagic stroke with residual UE hemiparesis, age 50–80, >6 months post stroke having completed all conventional therapy, able to perform ¼ range grasp (finger flexion) with at least two fingers with the wrist held passively in a neutral position (requirement for use of training orthotic), ability to stand unsupported for 5 minutes with contact guard. Subjects were excluded with acute serious medical conditions, dense flaccid hemiparesis, and fixed wrist or finger contractures in the paretic limb that precluded active movement with the Saeboflex device. Nine additional subjects were excluded from fMRI testing, but not the full project, due to claustrophobia, body size too large for fMRI bore and/or magnetic metal implants in the head, neck and torso. At baseline, no subject was able to independently grasp, transport and release a 6-inch diameter ball and all reported that they could not use the hand in activities of daily life. See Table 1 for subject characteristics by group.

**Table 1:** **Subject characteristics**

**Subject characteristics:**	**SAEBO group** ***n*** **= 14**	**COMBO group** ***n*** **= 13**
Baseline FM	23 ± 12.1	18.7 ± 9.1
Gender	9 Males/5 Females	7 Males/6 Females
Mean age	57	56
Mean time since stroke	3.1 years	5.3 years
Side of stroke (hemisphere)	10 Right/4 Left	4 Right/9 Left

### Ethics statement

This study was performed with the approval of the University of Maryland Baltimore and the Baltimore Veterans Administration IRB boards in compliance with the Helsinki Declaration. All subjects provided written informed consent. Both IRB boards approved the consent process.

### Impairment/functional measures

Impairment and functional measures were collected at baseline, after phase 1 of training (six-weeks), after phase 2 of training (12-weeks) and after retention (18-weeks) for all 26 subjects. Measures, all of which had reliability and validity testing on chronic stroke survivors included: 1. FM, a measure of impairment, [16,17], 2. Modified Wolf Motor Function Test (MWMFT) a timed functional measure modified for a moderately impaired population [18], 3. Box and Blocks (BB) a measure of hand function [19,20], 4. University of Maryland Arm Questionnaire for Stroke (UMAQS), assessing functional use of the arm in daily tasks [11,21], and 5. Modified Ashworth Scale (MAS), a measure of tone [22,23]. Testers were blinded to group assignment [11,18,21,24].

### Functional imaging

#### Task-based

fMRI was collected at baseline, mid-point and after training at 12-weeks on the 17 subject subset and was performed using a 3 T scanner (Philips, Eindhoven, The Netherlands). Briefly, 170 coronal blood oxygenation-level dependent (BOLD) weighted volumes (echo planar imaging sequence, TE = 40 ms, TR = 3 sec, 35–39 slices, slice thickness 5 mm, resolution 2 mm × 2 mm) covering the entire brain were acquired during movement of the NP and then the P arm/hand. For each arm, scans were obtained during 8 cycles of rest (10 volumes) followed by movement (8 volumes) performed in response to an auditory cue given via headsets once every three seconds. At the end of the 8 cycles, 26 volumes were added to allow for removal of linear trends in the data. For arm movement, the arm was strapped to a device that provided limited gravity support and allowed elbow flexion/extension in one (sagittal-transverse) plane within a defined range of motion from 45° relative to standard anatomical position to 60-75° depending on the participant’s paretic arm movement ability. While not ideal for reflecting proximal muscle control, (given that shoulder movement produced unacceptable head motion artifacts), this paradigm was still more consistent with neural control for proximal vs. distal control. The excursion of range was controlled by physical stops and fiber optic signals allowed testers to confirm movement through the full excursion had occurred. In addition to stabilizing with foam padding, the elbow device minimized head movement by preventing direct contact between trunk and arm and limiting load transmission. Each subject’s individual range of motion for the P arm was applied to the NP arm and subsequently kept constant to avoid a change of movement excursion causing differences in activation patterns over time. For hand movement, a custom-made hand orthotic (made by Saebo Inc. patterned off the Saeboflex device) was used on the paretic hand that allowed finger flexion around a ball fixed to the palm of the device. The degree of finger flexion was limited by the device, adjustable for the subject’s capability and maintained identical for repeated scans. Compliance with the protocol and the presence or absence of mirror movements and head motion was assessed through a video monitor using two cameras (head and arms). Elbow and hand testing took place separately with elbow preceding hand. One arm was tested at a time with the NP tested first. A T1-weighted image set (3D-MPRAGE, resolution 1 × 1 × 1 mm^3^) was acquired for anatomical localization.

Data were processed using Brain Voyager (Brain Innovation BV, Maastricht, The Netherlands). Standard protocols, including correction for slice timing differences, head motion (< 3 mm in any coordinate) and normalization to the Talairach coordinate space using manually selected landmarks were used. Successful spatial registration was verified for each individual. All image data from participants with left-sided lesions were flipped about the mid-sagittal plane, so the affected hemisphere was always on the right.

Analysis of functional maps was based on the hypothesis that assumed differential modulation of brain activity by COMBO versus SAEBO. A fixed-effects analysis identified regions demonstrating different levels of activation at baseline versus the 12-week time point, (p < 0.05, Bonferroni-corrected) within each group providing several brain regions of interest. Mean beta weight values of all voxels in a region of interest were then computed for each subject and time point (0, 6 and 12-weeks). These values were entered into a repeated measures ANOVA analysis to detect significant difference in the time course between groups, i.e., the interaction group × time. Using their Talairach coordinates and the Talairach Daemon (http://www.talairach.org/) regions were attributed to Brodmann areas (BA). A laterality index was computed based on beta weights in predefined volumes of interest (VOI) using the formula LI = (b_right_ – b_left_)/(|b_right_| + |b_left_|). Volumes of interest (BA 4 and BA 6) were created using the Talairach map included in BrainVoyager, a single subject-based atlas of cortical VOIs. The VOIs were inflated (“Dilate” function in BrainVoyager applied three times) to honor the anatomical variability between subjects.

### Training

After initial screening, subjects were randomized to the combination (COMBO) group or the unilateral task-oriented arm training only (SAEBO) group. The interventions were time-matched with both groups receiving two six-week training sessions that took place 3 times per week lasting approximately one hour. In phase 1 of training, subjects in COMBO received progressive bilateral arm training with the BATRAC Tailwind device (Encore Path Inc.). The unilateral (SAEBO) group received task-oriented training with the P arm assisted by the Saeboflex hand orthosis. In Phase 2, both groups received unilateral training described below. The elements of non-progressive BATRAC training are reported in previous studies [10-12,21,24]. The arms are moved simultaneously (inphase) and alternating (antiphase) for four 5-minute bouts. During week one, the excursion for each limb and the preferred bilateral frequency of moving the handles to and fro in the horizontal plane was determined. Progression was individualized for each participant, for frequency and movement against gravity. Frequency was progressed every week for training bouts 1 (inphase) and 2 (antiphase). For bouts 3 and 4 participants moved at their preferred speed but with an increase in grade which was determined weekly by the highest a participant could move through at least the original excursion continuously for 5 minutes. The excursion of the P arm was increased as tolerated. All progressions were recorded for individual participants. Training in this phase took place in the seated position. The emphasis of this training was to practice basic arm transport and was not conducted in the functional context of standing.

Unilateral training (phase 2 for COMBO and both phases for SAEBO group) included a standardized protocol of motor retraining with the Saeboflex device. A Saeboflex device was fit for each subject by the first author (trained by Saebo Inc. in device fitting). All training occurred in standing, using specialized training equipment (modified balls, buckets, adjustable targets, and adjustable horizontal poles) available through Saebo Inc. lasting one hour. Six functional reaching tasks were designed with different grasps and hand/arm orientation. Tasks included shoulder flexion (forward reach and release tasks), shoulder abduction and external rotation ( lateral reach tasks) and combinations of forearm positions of supination and pronation during reaching – all key movements that are challenging after stroke and are part of daily living activities and therefore were also practiced in standing. Thirty repetitions for each task were completed and progressed in number and speed given individual participant tolerance.

### Analysis of functional data

To assess differences between the groups at each post-training measure with respect to multiple functional measures, we used a MANOVA analysis. Outcomes in the MANOVA were the FM, MWMFT, BB, UMAQS, and MAS. After finding global significance based on the MANOVA, we fit mixed effects longitudinal regression models for each outcome separately, based on assessments made at all four time points. These models incorporated a random subject effect to account for the within-subject correlations. Using specified contrasts from this model we assessed whether there were statistically significant changes within groups and statistically significant differences between the groups with respect to changes in each measure. All p-values reported are two-sided. Analyses were performed using SAS 9.2.

## Results

### Functional outcomes

At baseline there were no statistically significant differences between the groups for any measure. Table 2 shows the results of baseline, 6-weeks, 12-weeks (post phase 2), and 18-weeks (retention) testing for the FM, MWMFT (time), UMAQS, BB and the MAS. Overall, there was a significant difference between the groups with respect to changes between baseline and 12-weeks (p = 0.018 based on a MANOVA test) in favor of COMBO. For this time range, the COMBO group also showed greater improvements than the SAEBO group in the MWMFT (time), UMAQS functional use questionnaire and both groups showed within-group changes in FM and BB. Neither group showed improvement in the MAS. At the retention measurement, there was also an overall significant difference between the groups (p = 0.0014 based on a MANOVA test) and at that time point, while the COMBO group showed better improvements in the MWMFT (time) score, and the UMAQS, the SAEBO group retained more improvements as measured by the FM and the BB.

**Table 2:** **Mean changes in functional measures, by training and time**

**Outcome**	**Baseline means**		**Mean change from baseline to 12-weeks**			**Mean change from baseline to retention time point**		
	**Mean**	**P-value for difference between groups** ^**1**^	**Mean (SD)**	**P-value for change** ^**2**^	**P-value for difference between groups** ^**2**^	**Mean (SD)**	**P-value for change** ^**2**^	**P-value for difference between groups** ^**2**^
Fugl Meyer		0.29			0.089			0.19
SAEBO	23.0 (12.1)		3.1 (5.3)	0.0013		3.8 (4.6)	0.0001	
COMBO	18.7 (9.1)		5.7 (2.4)	<0.0001		1.8 (3.8)	0.059	
Wolf (time)		0.17			<0.0001			0.011
SAEBO	78.2 (24.0)		−2.0 (6.6)	0.21		−4.9	0.0064	
COMBO	89.9 (9.1)		−13.9 (9.7)	<0.0001		−12.4	<0.0001	
Box and Block		0.56			0.99			0.12
SAEBO	3.3 (5.7)		1.8 (3.3)	0.0067		1.5 (2.8)	0.020	
COMBO	2.3 (3.3)		2.0 (3.8)	0.0089		0.0 (1.8)	0.95	
UMAQS		0.49			0.051		0.0069	0.015
SAEBO	16.4 (5.6)		3.8 (5.3)	0.0039		3.5 (7.7)	<0.0001	
COMBO	15.1 (4.4)		8.1 (7.6)	<0.0001		7.0 (7.4)		
Mod. Ashworth		0.27			0.75			0.44
SAEBO	1.6 (1.0)		0.0 (0.8)	0.76		0.0 (0.9)	0.76	
COMBO	2.1 (1.4)		−0.2 (1.1)	0.48		−0.4 (1.7)	0.21	

^1^Based on a two-sample t-test.

^2^Based on a mixed effects model using data from all four time points.

### Functional imaging

**Figure 1:**
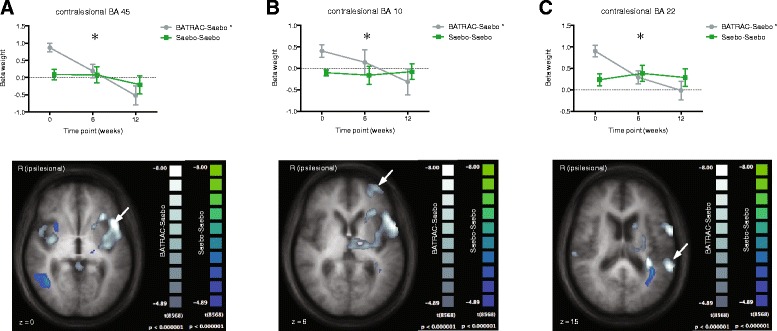
**Brain activation during paretic elbow movement was analyzed according to hand movement (see Figure** 2 **): A-C (BA45, 10 and 22 respectively) were down-regulated during COMBO (p < 0.05) but unaffected by two bouts of SAEBO training (interaction group × time p < 0.05).**

**Figure 2:**
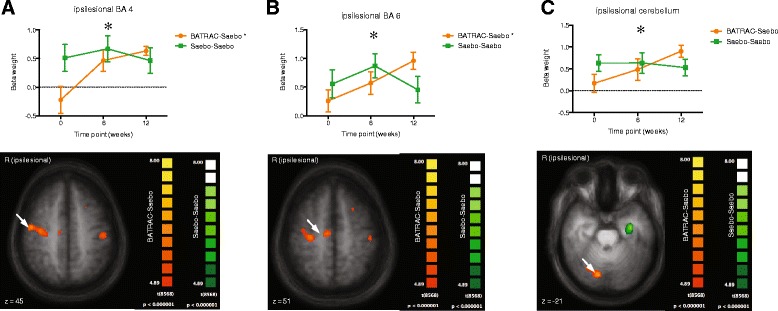
**Brain activation during paretic hand movement: Significant group effects for primary motor (A), premotor (B) and cerebellum (C) increased for COMBO with no change in SAEBO.**

During P elbow movement in BA45, 10 and 22 activity decreased in the COMBO group but did not significantly change in SAEBO subjects (Figure 1A-C). A supplementary analysis was computed to test whether the current sample of participants receiving BATRAC training replicated the results of previous studies of 6 weeks training [12,14]. We computed the contrast “post – pre-BATRAC” for the elbow scan using a fixed effects model (p < 0.05, Bonferroni-corrected). Similar to previous cohorts, BATRAC lead to an increase in activation in the ipsilesional premotor cortex (Talairach coordinates 27/16/58) and bilateral cerebellar hemispheres (28/-71/-35 and −16/-71/-33). Additional activation was found in the posterior cingulum (±7/-56/24) (Figure 2).

**Figure 3:**
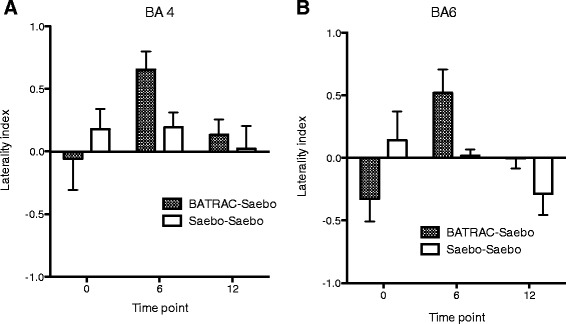
**Laterality index of activation in motor cortices during paretic hand movement demonstrates a shifting of activation towards the ipsilesional hemisphere after BATRAC training (interaction group × time for BA 4 p = 0.13 (A), for BA 6 p = 0.012 (B)).**

During paretic hand movement, several brain regions showed significant activation change after the 12 week intervention (COMBO or SAEBO) as compared with baseline (fixed effects model, Bonferroni-corrected p < 0.05). The time course of activation (baseline, 6-weeks, 12-weeks) for each of these regions was then compared between groups. Significant group effects (repeated measures ANOVA) were found for the contralateral (ipsilesional) primary motor area (BA4, p = 0.033), premotor cortex (BA6, p = 0.047) and cerebellum (p = 0.035). Activation of these areas was increased in the COMBO group, but did not change in the SAEBO group (Figure 2A-C). The laterality index of BA4 and BA6 increased after BATRAC and then decreased after SAEBO training, while showing no change in the SAEBO group. (Figure 3A,B). Only for BA6, was a significant between-group difference observed (repeated measures ANOVA, interaction group × time: p = 0.012). For primary motor cortex (BA4) between group comparisons were not significant (p = 0.13). Other regions increased (ipsilateral = contralesional BA32, 47, 20) or decreased (contralateral BA31, ipsilateral BA39) their activation in the SAEBO group, but showed no modulation in the COMBO group (between group differences p < 0.05).

## Discussion

In this randomized controlled study we show that a sequential rehabilitation approach combining bilateral and unilateral task oriented training improved arm and hand function more than unilateral task oriented training alone in a sample of participants with moderate to severe chronic hemiparesis. Although the time-matched control group of unilateral task oriented training also showed improvement, overall, the benefit for COMBO was greater as evidenced by significant gains in more of the functional outcomes, and larger effect sizes at post training and retention time points.

There is currently debate over which approach may be best for retraining upper extremity function. This debate may be better framed by the question, “which approach should be used for a certain level of functional ability”. We suggest that for the level of participant in a FM range of 9 – 35 starting with a proximal bilateral approach is beneficial for subsequent whole arm task oriented training that includes both transport and grasp/release components at least in the chronic stage. These participants with substantial residual hemiparesis may need to develop proximal control prior to progressing to more skilled unilateral upper extremity task training. In line with our conceptual model, one plausible mechanism may be that the bilateral training focus on proximal control recruits additional neural pathways such as uncrossed corticospinal or bilateral sub-cortical pathways such as rubrospinal that are then available to support the crossed corticospinal pathways needed to accomplish unilateral distal hand function. In addition, the bilateral training may disinhibit the crossed corticospinal pathways further priming the system for subsequent recruitment. Without building up a back-up system first or providing the additional priming, the unilateral distal focus may be unable to recruit adequate additional crossed corticospinal pathways.

The combination of training paradigms chosen for this study is only one option for sequential training. This study cannot speak to which sequence or duration of therapy cycles are optimal. Studies on duty cycle are still needed to determine exercise prescription guidelines and changing methodology should determine an individual’s progress rather than a prescribed time period such as used in this study. We suggest that a sequential progressive approach is better than one approach alone particular for these participants with limited proximal and distal control. We selected BATRAC because it focuses on re-learning basic shoulder/elbow control necessary for reaching. We used the Saeboflex device in the unilateral task oriented training in Phase 2 as it permitted the active involvement of the hand in grasp/release activities that would have been impossible without the aid of the device given the level of severity of the subjects. This combination proved to be well tolerated and efficacious in this sample.

The results of fMRI for P hand movement in our subset of subjects, suggest a differential modulation of control networks in the two groups. Ipsilesional primary motor and premotor cortices both increased their activity during COMBO but not during SAEBO therapy Hence, bilateral-unilateral sequential training ultimately increased the ipsilesional cortical involvement in the control of the contralateral hand. Plotting the strength of activation over time before and after each therapy cycle, shows a nearly linear increase of activation in these areas during 6 weeks of BATRAC followed by 6-weeks of SAEBO. The group that starts with SAEBO instead demonstrates some increase during the first 6 weeks but then a reduction during the second bout of SAEBO. This suggests that while COMBO can fully exploit the capacity of motor cortices and cerebellum for therapy-related upregulation, the changes induced by SAEBO reach a plateau after 6-weeks that cannot be overcome by more of the same therapy. The laterality index for the motor cortices shows a shift of activation towards the ipsilesional hemisphere during BATRAC followed by a return to bilateral activation during SAEBO. Ipsilesional shifting of activation has been observed after different types of effective training interventions [25-27] and has demonstrated a correlation with functional improvement. The return to bilateral activation, while not anticipated, may be secondary to the complexity of the movement tasks during the SAEBO phase of training. COMBO training, in contrast to SAEBO, also recruited the ipsilesional cerebellum. Cerebellar recruitment – however, contralateral to the lesion – has been suggested to differentiate patient with high versus low degrees of motor arm/hand recovery [28]. The ipsilesional cerebellum is mostly interconnected with the contralesional hemisphere. Recruitment of activation here, therefore, suggested that the intact hemisphere is undergoing functional changes related to the training. This is consistent with our prior work showing recruitment of the contralesional motor cortices after BATRAC training [12,14]. SAEBO training although beneficial for arm function as well, seems to use a different mechanism that is not reflected in ipsilesional shifting of activation in motor cortices but uses other cortical networks. During unimodal SAEBO training, several brain regions showed a continuous increase in activity. These areas were in the posterior cingulate cortex (BA31), inferior temporal gyrus (BA20) and the inferior frontal gyrus (BA47). The two latter ones were only enhanced after the second bout of SAEBO training suggesting a threshold effect of training intensity. While the cause of the changes in these areas – that are involved in visual processing and recognition (BA20), language processing (BA47) and awareness (BA31) – remains unresolved, one must consider that these changes were associated with the less effective (unimodal) intervention, hence, they may have less behavioral relevance.

During elbow movement several regions showed a decrease during COMBO. This is in contrast to most studies reporting increases in activation after rehabilitative training especially in the ipsilesional cortex [12,14,29,30]. During successful recovery (independent of the effects of specific therapies) activation that is spread across both hemispheres and exceeding that of healthy controls early after the stroke, is subsequently reduced and focuses on motor cortical region in the ipsilesional hemisphere [31]. Similar focusing of brain activation in certain brain regions could be a mechanism explaining the effectiveness of COMBO, although this is highly speculative given regions showing this effect in our data do not belong to classical motor (but language) networks. It is plausible that the length of training in this study has led to the focusing relative to our previous studies. It must be noted that based on our principal hypothesis here, we analyzed only regions that showed within-group differences between baseline and the 12-week time point, i.e., in case of COMBO after BATRAC and SAEBO interventions. Changes induced by BATRAC alone were reported before [12,14] and were confirmed here by an analysis contrasting activation patterns before and after BATRAC.

### Study limitations

We would like to acknowledge limitations of this current study. We recognize that we do not have an equal number of subjects with right and left sided lesions in each group, therefore we cannot definitively rule out an impact of side of stroke on treatment outcomes. Furthermore given that all subjects did not qualify for imaging, we do not have imaging data for all subjects for which we have functional outcome data.

## Conclusion

Combining a proximal bilateral training program sequentially with unilateral task oriented training may be more effective to facilitate gains in arm and hand function in those with more severe chronic paresis post stroke compared to unilateral task oriented training alone.

## Abbreviations

COMBO: Combination bilateral arm training and task oriented training
SAEBO: Task oriented training alone
fMRI: functional magnetic resonance imaging
P: Paretic
NP: Nonparetic
FM: Fugl-Meyer UE Test
MWMFT: Modified Wolf Motor Function Test
UMAQS: University of Maryland Arm Questionnaire for Stroke
MAS: Modified ashworth scale
BOLD: Blood oxygenation-level dependent
BA: Broadman areas
VOI: Volumes of interest

## References

[1] Butler AJ, Page SJ (2006). Mental practice with motor imagery: evidence for motor recovery and cortical reorganization after stroke. Arch Phys Med Rehabil.

[2] Taub E, Uswatte GMA (2000). Constraint-induced movement therapy and massed practice. Stroke.

[3] Taub E, Uswatte G, Pidikiti R (1999). Constraint-induced movement therapy: a new family of techniques with broad application to physical rehabilitation-aclinical review. J Rehabil Res Dev.

[4] Taub E, Wolf SW (1997). Constraint induced movement techniques to facilitate upper extremity use in stroke patients. Top Stroke Rehabil.

[5] Page SJ, Sisto S, Johnston MV, Levine P (2002). Modified constraint-induced therapy after subacute stroke: a preliminary study. Neurorehabil Neural Repair.

[6] Liepert J, Miltner WH, Bauder H, Sommer M, Dettmers C, Taub E, Weiller C (1998). Motor cortex plasticity during constraint-induced movement therapy in stroke patients. Neurosci Lett.

[7] Bonifer NM, Anderson KM, Arciniegas DB (2005). Constraint-induced movement therapy after stroke: efficacy for patients with minimal upper-extremity motor ability. Arch Phys Med Rehabil.

[8] Lum PS, Burgar CG, Shor PC, Majmundar M, Van der Loos M (2002). Robot-assisted movement training compared with conventional therapy techniques for the rehabilitation of upper-limb motor function after stroke. Arch Phys Med Rehabil.

[9] Hesse S, Werner C, Pohl M, Rueckriem S, Mehrholz J, Lingnau ML (2005). Computerized arm training improves the motor control of the severely affected arm after stroke: a single-blinded randomized trial in two centers. Stroke.

[10] McCombe Waller S, Whitall J (2005). Hand dominance and side of stroke affect rehabilitation in chronic stroke. Clin Rehabil.

[11] Whitall J, McCombe Waller S, Silver KHC, Macko RF (2000). Repetitive bilateral arm training with rhythmic auditory cueing improves motor function in chronic hemiparetic stroke. Stroke.

[12] Luft AR, McCombe-Waller S, Whitall J, Forrester LW, Macko R, Sorkin JD, Schulz JB, Goldberg AP, Hanley DF (2004). Repetitive bilateral arm training and motor cortex activation in chronic stroke: a randomized controlled trial. Jama.

[13] Luft AR, Waller S, Forrester L, Smith GV, Whitall J, Macko RF, Schulz JB, Hanley DF (2004). Lesion location alters brain activation in chronically impaired stroke survivors. Neuroimage.

[14] Whitall J, Waller SM, Sorkin JD, Forrester LW, Macko RF, Hanley DF, Goldberg AP, Luft A (2011). Bilateral and unilateral arm training improve motor function through differing neuroplastic mechanisms: a single-blinded randomized controlled trial. Neurorehabil Neural Repair.

[15] Luft AR, Macko RF, Forrester LW, Villagra F, Ivey F, Sorkin JD, Whitall J, McCombe-Waller S, Katzel L, Goldberg AP, Hanley DF (2008). Treadmill exercise activates subcortical neural networks and improves walking after stroke. a randomized controlled trial. Stroke.

[16] Berglund K, Fugl-Meyer A (1986). Upper extremity function in Hemiplegia. Scand J Rehabil Med.

[17] Filiatraut J, Arsenault A, Dutil E, Bourbonnais D (1991). Motor function and activities of daily living assessments: a study of three tests for persons with Hemiplegia. Am J Occup Ther.

[18] Whitall J, Savin DN, Harris-Love M, Waller SM (2006). Psychometric properties of a modified wolf motor function test for people with mild and moderate upper-extremity hemiparesis. Arch Phys Med Rehabil.

[19] Platz T, Pinkowski C, van Wijck F, Kim IH, di Bella P, Johnson G (2005). Reliability and validity of arm function assessment with standardized guidelines for the Fugl-meyer test, action research arm test and box and block test: a multicentre study. Clin Rehabil.

[20] Siebers A, Oberg U, Skargren E (2010). The effect of modified constraint-induced movement therapy on spasticity and motor function of the affected arm in patients with chronic stroke. Physiother Can.

[21] Bovend’Eerdt T, Dawes H, Johansen-Berg H, Wade D (2004). Evaluation of the modified jebsen test of hand function and the University of Maryland arm questionnaire for stroke. Clin Rehabil.

[22] Brashear A, Zafonte R, Corcoran M, Galvez-Jimenez N, Gracies JM, Gordon MF, McAfee A, Ruffing K, Thompson B, Williams M, Lee CH, Turkel C (2002). Inter- and intrarater reliability of the ashworth scale and the disability assessment scale in patients with upper-limb poststroke spasticity. Arch Phys Med Rehabil.

[23] Katz RT, Rovai GP, Brait C, Rymer WZ (1992). Objective quantification of spastic hypertonia: correlation with clinical findings. Arch Phys Med Rehabil.

[24] McCombe Waller S, Whitall J (2004). Fine motor control in adults with and without chronic hemiparesis: baseline comparison to nondisabled adults and effects of bilateral arm training. Arch Phys Med Rehabil.

[25] Boyd LA, Vidoni ED, Wessel BD (2010). Motor learning after stroke: is skill acquisition a prerequisite for contralesional neuroplastic change?. Neurosci Lett.

[26] Michielsen ME, Smits M, Ribbers GM, Stam HJ, van der Geest JN, Bussmann JB, Selles RW (2011). The neuronal correlates of mirror therapy: an fMRI study on mirror induced visual illusions in patients with stroke. J Neurol Neurosurg Psychiatry.

[27] Bhatt E, Nagpal A, Greer KH, Grunewald TK, Steele JL, Wiemiller JW, Lewis SM, Carey JR (2007). Effect of finger tracking combined with electrical stimulation on brain reorganization and hand function in subjects with stroke. Exp Brain Res.

[28] Small SL, Hlustik P, Noll DC, Genovese C, Solodkin A (2002). Cerebellar hemispheric activation ipsilateral to the paretic hand correlates with functional recovery after stroke. Brain.

[29] Johansen-Berg H, Dawes H, Guy C, Smith SM, Wade DT, Matthews PM (2002). Correlation between motor improvements and altered fMRI activity after rehabilitative therapy. Brain.

[30] Schaechter JD (2004). Motor rehabilitation and brain plasticity after hemiparetic stroke. Prog Neurobiol.

[31] Feydy A, Carlier R, Roby-Brami A, Bussel B, Cazalis F, Pierot L, Burnod Y, Maier MA (2002). Longitudinal study of motor recovery after stroke: recruitment and focusing of brain activation. Stroke.

